# Uncovering Latent Cognitive, Metacognitive, and Neural Bases of Problem-Solving Deficits in Children with Developmental Dyscalculia

**DOI:** 10.1523/JNEUROSCI.2200-25.2026

**Published:** 2026-09-21

**Authors:** Oliver H. M. Lasnick, Yunji Park, Percy Mistry, Vinod Menon

**Affiliations:** ^1^Department of Psychiatry & Behavioral Sciences, Stanford University, Stanford, California 94305; ^2^Department of Neurology & Neurological Sciences, Stanford University, Stanford, California 94305; ^3^Stanford Neurosciences Institute, Stanford University, Stanford, California 94305; ^4^Symbolic Systems Program, Stanford University, Stanford, California 94305

**Keywords:** developmental dyscalculia, latent cognitive mechanisms, multivariate analysis, neural decoding

## Abstract

Developmental dyscalculia (DD) affects 7–10% of children and is characterized by persistent difficulties in arithmetic problem-solving, which depends on the dynamic interplay between cognitive and metacognitive control processes governing strategy execution and selection. Conventional behavioral and neuroimaging approaches do not simultaneously quantify these latent processes and link them to distributed neural mechanisms, leaving the neurocognitive and metacognitive underpinnings of DD insufficiently characterized. We integrated Bayesian computational modeling with whole-brain decoding to uncover cognitive, metacognitive, and neural bases of problem-solving deficits in DD using a sample of 68 children aged 8–10 years (38 female). Children with DD showed deficits across all problem-solving strategies (counting, retrieval, and decomposition), indicating reduced strategy execution efficiency. They also exhibited significant metacognitive impairments: longer baseline switching times between strategies, reduced sensitivity to problem difficulty, and lower adaptivity in selecting optimal strategies. Whole-brain Elastic Net decoding accurately distinguished DD from TA peers based on distributed activation patterns across frontoparietal cortex, hippocampus, and ventral-visual regions, whereas univariate approaches did not. Linear multivariate brain–behavioral analyses and nonlinear transformer-based predictions converged in showing that these distributed signatures predicted counting efficiency and switching sensitivity. Notably, brain–behavior relations reversed in direction between groups, positive in TA children but negative in DD, most strikingly in the hippocampus. These findings demonstrate that arithmetic difficulties in DD arise from deficits in both strategy execution efficiency and metacognitive control, reflecting an altered mapping between distributed neural activity, and highlight the power of integrating computational modeling with brain-wide decoding to understand learning disabilities.

## Significance Statement

Developmental dyscalculia affects 7–10% of children, yet underlying mechanisms remain poorly understood. We integrated Bayesian computational modeling with whole-brain decoding to demonstrate that children with dyscalculia exhibit deficits in both strategy execution efficiency (counting, retrieval, decomposition) and metacognitive control (strategy switching, adaptive strategy selection). Using brain-wide decoding, we identified distributed neural signatures across frontoparietal, hippocampal, and visual regions that distinguished dyscalculia from typically developing children, patterns undetectable by conventional approaches. These neural signatures predicted strategy selection and execution. Our findings demonstrate that developmental dyscalculia reflects cognitive and neural dysfunction across multiple processes, highlighting the power of combining computational modeling with multivariate neuroimaging analysis for understanding individual variability in learning disabilities.

## Introduction

Developmental dyscalculia (DD) affects 7–10% of school-age children and is characterized by persistent deficits in arithmetic problem-solving that significantly impair academic achievement and daily functioning (Barbaresi et al., 2005; Geary, 2011). Successful problem-solving depends on the dynamic interplay between cognitive processes, such as executing retrieval or backup procedural strategies, and metacognitive control processes that guide adaptive strategy selection and flexible switching across trials (Siegler, 1996, 2007; Siegler et al., 1996). Yet the cognitive, metacognitive, and neural sources of the problem-solving difficulties in children with DD remain insufficiently understood.

Behavioral studies based on self-reported and observational strategy assessments have documented that children with DD draw on the same strategy repertoire as typically achieving (TA) peers: counting, fact retrieval, and decomposition (e.g., solving 3 + 8 by reformulating as [2 + 8] + 1). However, they tend to make more errors in executing these strategies (Russell and Ginsburg, 1984; Geary et al., 1992; Geary et al., 2012). Beyond strategy execution, children with DD rely more heavily on inefficient backup counting strategies (Ostad, 1997; Geary et al., 2004, 2012) and are less adaptive in strategy selection (Torbeyns et al., 2004; Peters et al., 2014), implicating impairments in the top-down process governing adaptive and flexible strategy selection across trials. Such processes reflect metacognitive control, the regulation of cognition through goal-setting and planning, as well as metacognitive monitoring, the evaluation of one's own performance (Nelson and Narens, 1990; Ohtani and Hisasaka, 2018).

These approaches have yielded valuable insights into strategy-level deficits in DD. However, self-reports and aggregate behavioral measures are not designed to quantify the latent cognitive and metacognitive control processes that unfold trial-by-trial, leaving the dynamic mechanisms driving performance differences poorly characterized. Only a handful of computational models have begun to uncover these latent processes and primarily in TA children (Siegler and Shipley, 1995; Supekar et al., 2021; Mistry et al., 2025).

Neuroimaging studies have reported atypical functional activation in children with DD during arithmetic problem-solving (Ashkenazi et al., 2012; Rosenberg-Lee et al., 2015; Peters and De Smedt, 2017), yet interpretation remains constrained by two gaps. First, few studies have directly modeled children's in-scanner problem-solving strategies, making it unclear whether atypical activation reflects disruptions in strategy execution or selection. Second, most studies rely on traditional univariate analyses that emphasize regional activity while overlooking distributed network coordination. Both limitations are underscored by Kwok et al. (2023), who observed clear behavioral impairments, but no atypical functional activation, in a well-characterized DD sample using conventional methods, highlighting the need for more sensitive and deeper computational approaches.

To address these gaps, we integrated advanced Bayesian computational modeling with multivariate neuroimaging to link specific cognitive and metacognitive control processes to distributed neural activity, uncovering the mechanisms underlying arithmetic deficits in children with DD. We analyzed a publicly available dataset from Kwok et al. (2023), comprising a relatively large sample of 65 children (30 DD, 35 TA) diagnosed through systematic screening by the Singaporean Ministry of Education (see Materials and Methods), affording greater statistical power than typical neuroimaging studies of DD.

Our first goal was to quantify the latent processes governing strategy execution and selection in children with DD using the Bayesian Model of Arithmetic Problem-Solving (BMAPS; Fig. 1; Supekar et al., 2021; Mistry et al., 2025). BMAPS estimates trial-by-trial probabilities of using counting, retrieval, and decomposition strategies, along with execution efficiency based on accuracy and reaction time patterns and yields intertrial metacognitive control parameters governing strategy selection, including switching time, switching sensitivity, and adaptivity. Our second goal was to identify distributed neural patterns that distinguish children with DD from TA children, using Elastic Net classification, a multivariate machine learning approach well suited for identifying distributed neural signatures (Zou and Hastie, 2005). Finally, employing a novel AI-based transformer model, we tested whether these distributed neural signatures predict the latent cognitive and metacognitive processes captured by BMAPS parameters, linking brain function to the computational mechanisms underlying strategy execution and selection in DD. Together, this integrated approach offers a new mechanistic account of the sources of numerical problem-solving deficits and individual variability in children with DD.

**Figure 1.**
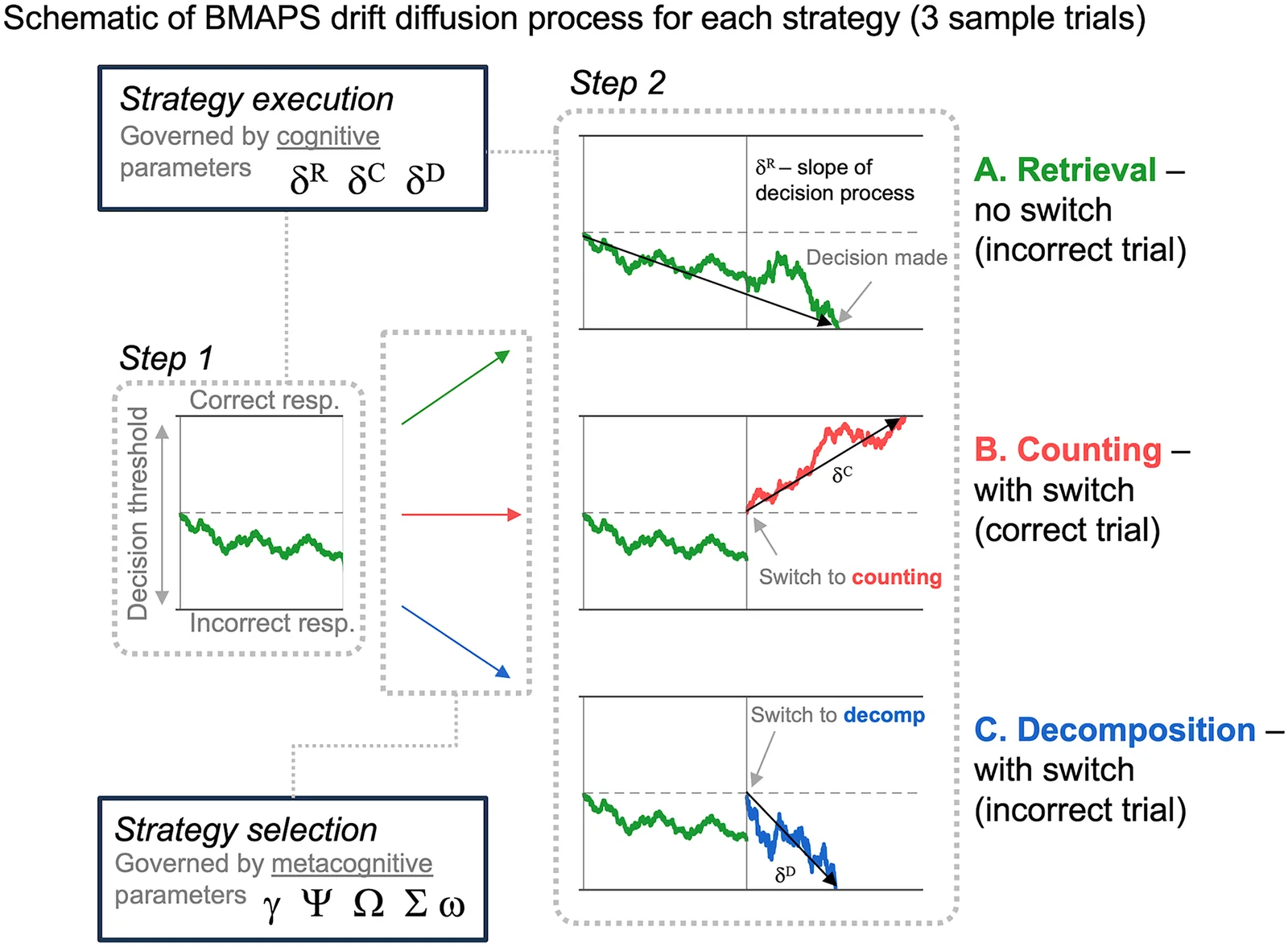
Schematic of Bayesian model of arithmetic problem-solving (BMAPS) framework. BMAPS models behavior as emerging from a latent mixture of drift diffusion models, each representing a distinct cognitive strategy—retrieval, counting, and decomposition—embedded within a hierarchical metacognitive framework. Strategy selection follows two steps: Step 1, an initial bias toward retrieval during which evidence accumulates (green trajectory) toward either a correct (top) or an incorrect (bottom) response and Step 2, potential mid-trial switch when retrieval fails to reach threshold. In such cases, a probabilistic switch may occur from retrieval (***A***) to continued retrieval, (***B***) counting, or (***C***) decomposition, with the new strategy continuing until the decision threshold is reached or the trial ends. BMAPS infers the strategy use on each trial by comparing likelihoods across strategy-specific parameterization, yielding both trial-level and individual-level measures of cognitive and metacognitive processes. Cognitive parameters include strategy-specific evidence accumulation efficiency—retrieval (δ^R^), counting (δ^C^), and decomposition (δ^D^). Metacognitive parameters include strategy switching adaptivity (Ψ), baseline switching time (γ), switching sensitivity to problem difficulty (ω), and switching uncertainty and variability (mean entropy Ω and standard entropy Σ). This sequential, within-trial modeling captures dynamic strategy adaptations that traditional self-report methods cannot detect. Figure adapted from Mistry et al. (2025).

## Materials and Methods

### Participants

We used the publicly available dataset from Kwok et al. (2023) on OpenNeuro (doi:10.18112/openneuro.ds004791.v1.0.0). The original sample included 68 children aged 8–10 years (*M* = 8.95 years, SD = 0.34; 38 female). Of these, 30 children were classified as having developmental dyscalculia (DD) based on the following criteria: they were either (1) identified by Singapore's Ministry of Education as having difficulty in math through screening at the beginning of primary school and subsequently referred to an intervention program or (2) scored in the bottom 10th percentile of the Test of Early Mathematics Ability (TEMA-3). Children who scored above the 25th percentile on the TEMA-3 were classified as typically achieving (TA, *N* = 38). We excluded three additional participants who were missing all data from either the arithmetic verification task or the matching task, resulting in a sample of 65 children (36 female; 28 with DD and 37 TA) for traditional behavioral and neuroimaging analyses. An additional two participants were missing one run from the arithmetic verification task, and these two were excluded from the BMAPS analyses, as the model could not be run with inconsistent data between participants. This resulted in 63 participants for BMAPS model parameter estimation (35 female, 27 DD, 36 TA). Additional details regarding sample characteristics and group classification are provided in Kwok et al. (2023).

### Experimental design and statistical analyses

#### Arithmetic verification task

In this task, participants were presented with an addition equation comprising two single-digit addends and a proposed solution that could be either correct or incorrect (e.g., 5 + 3 = 7, incorrect trial). They were instructed to determine whether the presented equation was correct or incorrect. Half of the trials presented a correct sum and the remaining half presented an incorrect sum that exceeded the true answer by 1 or 2. One-third of trials were considered “Large” problems (solution greater than 10), one-third were “Small” problems (solution less than or equal to 10), and one-third were “Plus1” problems, where one addend equaled 1 (e.g., 2 + 1 = 3). Each run consisted of 36 trials presented in block design, with two runs presented to each participant.

#### Matching task

In this task, participants were presented simultaneously with two stimuli side-by-side and instructed to determine whether the two stimuli matched or not. Half of the trials had matching stimuli, and the other half had non-matching stimuli. There were three types of stimuli: numbers, shapes, or faces. In the “Number” condition (one-third of trials), children were presented with a symbolic number and a set of dots and asked to judge whether the quantities the two stimuli represented were the same. Incorrect trial stimuli differed from one another in quantity by two, three, or four (e.g., a set of six dots could be presented with the digit “3”). In the “Shape” condition (one-third of trials), they were presented with two regular geometric shapes (e.g., a circle and a square) and asked to judge whether the shapes were the same. There were nine total shapes. In the “Faces” condition (one-third of trials), they were presented with two front-facing human faces derived from 3D models and asked to judge whether the faces were the same. There were nine total “base” faces. For incorrect trials, one of the base faces was paired with an altered version of itself, with either small or large differences. Each run consisted of 54 trials presented in block design, with two runs presented to each participant.

#### Behavioral analysis

To compare differences in task performance between the DD and TA groups cross different conditions, we conducted independent-samples *t* tests for both accuracy and RT separately in both tasks (arithmetic, matching) and across all task conditions separately (Supplementary Fig. S1). All behavioral analyses were conducted using a Bayesian statistical framework as implemented in JASP software v0.19.3 (https://jasp-stats.org/) to quantify the strength of evidence for group differences and accurately assess the presence or absence of effects across task conditions. We expected that the DDs would have lower accuracy and higher RT than the TAs for all arithmetic conditions (especially the Large problems) and for the number-matching condition.

Statistical significance was determined using Bayes factors (BF_10_) which allow evaluation of the presence or absence of evidence for the alternative hypothesis (Keysers et al., 2020). BF_10_ values exceeding 3 indicate evidence for the test hypothesis, while values between 0.33 and 3 suggest no decisive evidence, and values lower than 0.33 indicate evidence for the null hypothesis. The strength of evidence also increases with BF_10_: values 3–10 indicate moderate evidence, 10–30 strong evidence, 30–100 very strong evidence, and values above 100 indicate extreme evidence (Faulkenberry et al., 2020). Effect size (*δ*) was also estimated from the posterior probability distribution as the difference between the group means divided by the standard deviation, analogous to Cohen's *d*.

#### Bayesian model of arithmetic problem-solving and strategy selection

To uncover cognitive variability underlying arithmetic problem-solving in children with DD, we employed a recently developed Bayesian model for arithmetic problem-solving (Mistry et al., 2025; BMAPS; Fig. 1) to model in-scanner arithmetic task performance. This model infers the likelihood of using counting, retrieval, and *decomposition* (i.e., breaking down addends into smaller components to simplify problem-solving, for example, solving 3 + 8 by splitting 3 into 1 and 2 to create a base-10 unit from 2 + 8) strategies on a trial-by-trial basis (Supplementary Tables S1, S2) by using the joint distribution of accuracy and RT, while accounting for the variability in item difficulty across trials.

BMAPS estimates individual-level cognitive and metacognitive control parameters related to strategy execution, such as strategy-specific drift rate (reflecting efficiency) and strategy selection parameters. The model estimates several key metacognitive control parameters: First, baseline (strategy) switching time refers to the average time required to switch strategies. Second, switching sensitivity indicates the extent to which switching time is influenced by item difficulty. Third, retrieval propensity represents the tendency to favor retrieval over other strategies. Lastly, adaptivity reflects the optimality of an individual's retrieval strategy selection, calculated from the correlation between their retrieval efficiency and actual strategy choices across trials. A full list of parameters is provided in Table 1.

**Table 1.** Definition of Bayesian model of arithmetic problem-solving (BMAPS) parameters

BMAPS parameter	Symbol	Definition
Retrieval drift rate	*δ* ^R^	Rate of evidence accumulation for retrieval
Counting drift rate	*δ* ^C^	Rate of evidence accumulation for counting
Decomposition drift rate	*δ* ^D^	Rate of evidence accumulation for decomposition
Retrieval propensity	*μ*	Tendency to favor retrieval over other strategies
Decision threshold	α	Amount of evidence required for a given decision
Baseline switching time^a^	γ	Time to switch strategies (baseline, across trials)
Switching sensitivity	*ω*	Modulates strategy switching time for a given trial based on ease of item retrieval
Counting boost	*κ*	Increase in efficiency for counting versus memory retrieval
Nondecision time^b^	*τ*	Portion of RT not dedicated to decision-making
L-R bias	*β*	Response bias for binary decision (0–1, 0.5 = no bias)
Adaptivity	*Ψ*	Optimality of strategy selection (across trials)
Mean entropy	*Ω*	Uncertainty in strategy choice (derived from latent probability of all strategies)
Standard entropy	*Σ*	Variability of mean entropy across trials

a Trial-level switching time is defined as the time at which an individual decides to switch away from the initial retrieval strategy; a longer switching time is expected with either a high switching sensitivity or if that trial's solution is easier to retrieve from memory (or if individual baseline switching time is higher).

b Includes time to encode the presented stimulus prior to beginning execution of the initial strategy; the remaining RT is assumed to reflect the actual strategy selection and decision-making processes.

In BMAPS, trial-level responses (choice—accurate or inaccurate—and reaction time) are modeled as a drift diffusion model (DDM). The response of an individual *i* on trial *t* is denoted as $x_{it}$ where $x_{it}=1$ if the response was correct and $x_{it}=-1$ if the response was incorrect. The response time is similarly denoted as $r_{it}.$ The choice coded response time is represented as $y_{it}=\;x_{it}r_{it}$. Here, $y_{it}$ is treated as a sample from random variable $Y_{it}$ which is distributed according to a Wiener distribution (Vandekerckhove et al., 2011; Wabersich and Vandekerckhove, 2014). The Wiener distribution describes the probability density function for $Y_{it}$, or in other words, the joint probability density function for the random variables $X_{it}$ and $R_{it}$. The Wiener distribution is a four-parameter distribution that describes the first passage time of the diffusion process, that is, the distribution of times taken for the diffusion process to first hit the positive (x=1) and negative (x=−1) decision boundaries. This is a canonical approach for modeling the joint distribution of choice and response time in two-choice processes (Wagenmakers, 2009; Ratcliff et al., 2016):

$$y_{i,t}\;∼\;\mathrm{Wiener}\;(\alpha_{i,t},\;\;\tau_{i,t},\;\;\beta_{i,t},\;\;\delta_{i,t}).\;(1)$$

Behavior is modeled as a mixture model of these latent strategies across trials. A latent value codes which strategy produces a response on any single trial: *S(t)*. The latent mixture DDM implemented can then be represented as follows:

$$y_{i,t}\;∼\;\mathrm{Wiener}\;(\alpha_{i,S(t)},\;\;\tau_{i,S(t)},\;\;\beta_{i,S(t)},\;\;\delta_{i,S(t)}).\;(2)$$

The selection of strategies is assumed to be dependent on a sequential process, with the first step modeling whether there is persistence of memory retrieval. This probability is modeled as a traditional 1-parameter logistic item response theory (1PL-IRT) model. Thus, for Step 1, the probability of an individual *i* selecting a memory retrieval strategy for an item *k* $\left(s_{ik}=M\right)$ depends on the individuals' proclivity toward memory retrieval $\mu_{iM}$ as well as how amenable the item is to be retrieved from memory $\eta_{kM}$ (this is measured at a group level) and follows the form of 1PL-IRT model. The $\eta_{kM}$ parameter can be interpreted as the ease of retrieval of item *k.* Importantly, this breaks down the propensity of retrieval into orthogonal individual and item level effects:

$$p(s_{ik}=M)=\frac{1}{1+e^{-(\mu_{iM}\;+\;\eta_{kM})}}.$$

In the second step, assuming that the result of the first step is to switch away from retrieval $\left(s_{ik}\neq M\right)$, a nominal response model (Bock, 1972) is considered. This step models the probability of selecting an alternate strategy *S* by simultaneously taking into consideration the remaining two alternate strategies (counting and decomposition), where the probability of counting depends on item level effects $\left(\eta_{kC}\right)$:

$$p(s_{ik}=C\;|\;s_{ik}\neq M)=\frac{1}{1+e^{-(\eta_{kC})}}.$$

This sequential process implies some switching point at which an individual might give up on the retrieval strategy and switch to an alternate strategy, in other words, the point at which the second step is initiated. Our model characterizes this switching time $\left(\xi_{ik}\right)$ as the point at which a decision is taken on whether to switch away from memory by individual *i* for item *k.* It is possible that the actual retrieval takes longer, but enough confidence has been gained by then to take a decision on whether to switch. This switching time will vary by individual, but it may further vary depending on the nature of the item. We propose that people may have some form of access to the ease of retrieval of an item $\eta_{kM}$ and their switching time may thus vary depending on a baseline switching time $\gamma_{i}$, switching sensitivity $\omega_{i}$, and ease of item retrieval $\eta_{kM}$:

$$\xi_{ik}=\gamma_{i}+\omega_{i}\;\eta_{kM}.$$

The memory retrieval process is characterized as a drift diffusion process, which posits that evidence accumulates over time resulting in a decision when a decision threshold is reached. The efficiency of evidence accumulation is characterized by a memory retrieval drift rate parameter $\left(\delta^{M}\right)$, with higher values characterizing faster and more accurate retrieval. The decision threshold parameter (*α*) captures the degree of confidence required to conclusively decide that a solution is retrieved from memory, with higher values characterizing slower but more accurate retrievals. The memory retrieval drift rate for an individual *i* for an item *k* is proposed as a combination of individual $\left(\delta_{i}^{M}\right)$ and item level effects:

$$\delta_{ik}^{M}=\frac{\delta_{i}^{M}}{1+e^{-\eta_{kM}}}.$$

Thus, for items which are considered very easy for retrieval (high positive values of $\eta_{kM}$), $\delta_{ik}^{M}\;\to \;\delta_{i}^{M}$, whereas for items considered very difficult for retrieval (high negative values of $\eta_{kM}$), $\delta_{ik}^{M}\;\to \;0$.

The counting process is also characterized as a drift diffusion process, with a combination of individual and item level effects. The counting strategy used in this model is assumed to be min counting (counting up from the larger addend)*.* For an item *k*, with the first addend presented being $n_{1k}$ and the second being $n_{2k}$, the drift rate for counting is modeled as follows:

$$\delta_{ik}^{C\;[\min]}=\frac{\delta_{i}^{C}}{\min(n_{1k},\;n_{2k})}.$$

The decomposition process can be characterized as a set of $n_{D}$ steps taken to break down the addition process. For instance, 8 + 7 may be broken down as 7 = 2 + 5; 8 + 2 = 10; 10 + 5 = 15, which involves $n_{D}=3$ steps:

$$\delta_{ik}^{D}=\;\frac{\delta_{i}^{D}}{n_{D}}.$$

Each of these steps is at least as difficult as a counting step, so we apply the constraint, $\delta_{i}^{D}\leq \;\delta_{i}^{C}$.

Additional details of the model are available in the supplementary material of Mistry et al. (2025).

To test whether children with DD exhibit deficits in the cognitive and metacognitive parameters of interest compared with their TA peers, we conducted independent-samples *t* tests within a Bayesian statistical framework for each parameter. Additional bivariate correlation analyses between BMAPS parameters and in-scanner arithmetic performance (accuracy and reaction times) were conducted for descriptive purposes only, to help readers contextualize the BMAPS parameters in relation to more intuitive performance measures. These correlations were not used for inferential conclusions.

As a control analysis to validate our BMAPS parameters, we examined problem size effects in strategy use. We first calculated the percentage of trials on which each strategy was used, separately for Small (sum ≤10), Medium (sum = 11–12), and Large (sum = 13–14) problems, based on sum values of the addition problems—consistent with the distinction between simple (small sum) and complex (medium and large sum) problems used by Geary et al. (2004)—and performed a chi-squared test across problem size and strategy bins.

#### fMRI data acquisition and preprocessing

Images from Kwok et al. (2023) were acquired on a Siemens 3 T MAGNETOM Prisma scanner using a 32-channel head coil (Siemens). A T1-weighted high-resolution structural image was acquired using the MPRAGE sequence (TR, 2,300 ms; TE, 2.29 ms; FOV, 204 mm; 0.9 mm isotropic voxels; and slice thickness of 0.94 mm). A T2*-weighted EPI functional image was acquired using the following parameters: TR, 1,000 ms; TE, 28 ms; FOV, 208 mm; flip angle, 40°; 2.5 mm isotropic voxels; and slice thickness of 2.5 mm.

Images were preprocessed and analyzed using SPM12. All structural images were skull stripped and segmented for coregistration. The preprocessing pipeline for functional data consisted of realignment, slice-timing correction, coregistration to each participant's individual structural T1-weighted image, and normalization to a standardized 2 mm Montreal Neurological Institute (MNI152) template in SPM and smoothing using a 6 mm full-width at half-maximum Gaussian kernel to decrease spatial noise. Translational motion (*x*, *y*, *z*) and rotational motion (pitch, roll, yaw) were calculated in millimeters based on the SPM12 realignment procedure. Mean framewise displacement of the participants did not exceed 0.6 mm in any single run.

#### fMRI data analysis: general linear model

Following preprocessing, a general linear model (GLM) implemented in SPM12 was used to compute individual-level task-related brain responses. Brain responses associated with correct trials for each task and condition of interest (Arithmetic task: Large, Small, and Plus1 conditions; Matching task: Number and Shape conditions) were modeled using boxcar functions aligned with the corresponding trial duration. These functions were convolved with a canonical hemodynamic response function and a temporal derivative to account for voxelwise variations in hemodynamic response latency. Additionally, both translational and rotational head motion parameters were included as covariates of no interest. We used correct trials only to maintain compatibility with Kwok et al. (2023) and because they better capture neural activity specific to arithmetic problem-solving and exclude processes unrelated to the task such as inattention, fatigue, or guessing that may be reflected in incorrect trials. Voxelwise contrast maps were generated for each participant for each task. For the arithmetic task, the primary contrasts of interest were Large + Small vs Plus1, as in Kwok et al. (2023), and Large versus Plus1, based on Matejko and Ansari (2019), both of which showed strong effects at the neural level. Although Small versus Plus 1 was not our primary contrast of interest, voxelwise contrast maps were generated to assess its significance. For the matching task, the contrast of interest was Number versus Shape, which allowed us to assess nonsymbolic and symbolic quantity processing, distinct from the visual processing of shapes.

Next, we performed a whole-brain one-sample *t* test to assess population-level within-subjects effects for each contrast of interest. A separate GLM was applied for the arithmetic and matching tasks. All statistical maps were masked with a gray matter mask, and significant clusters were thresholded with voxelwise FDR correction at *p* < 0.01 following Liu et al. (2025). Two binary masks derived from the identified significant voxels were selected for the subsequent EN decoding procedure. Masks were generated from the Large > Plus1 contrast from the arithmetic verification task and the Number > Shape contrast from the matching task. The Number > Shape mask was included because this contrast captures number-specific visual and semantic processing relevant to early arithmetic problem-solving stages and encompasses a broader set of numerical cognition-related regions than the Large > Plus1 mask alone. This approach restricted the voxel pool used for classification while preserving features relevant to arithmetic processing. The union of these masks was taken and used for subsequent decoding analysis.

#### Elastic Net decoding analysis

To test whether functional response patterns across distributed brain regions can distinguish children with DD from TA controls, we implemented an Elastic Net (EN) classifier using the python package glmnet_python (Balakumar, 2016) with leave-one-out cross-validation (LOOCV). This classifier integrates feature elimination from the least absolute shrinkage and selection operator (LASSO) method and feature coefficient reduction from ridge regression, resulting in shrinking the value of coefficients for model features that are relatively unimportant (Russell and Ginsburg, 1984; Geary, 1990; Geary et al., 2012).

Prior to decoding, we identified the task contrast and feature mask to be used. Among the arithmetic contrasts examined (Large + Small* > *Plus1, Large* > *Plus1, and Small* > *Plus1), Large* > *Plus1 produced the strongest and most widespread activation across frontoparietal and temporal regions (Supplementary Table S3) and corresponded to the condition with the most robust behavioral group difference; Small* > *Plus1 yielded no significant activation. Consistent with Kwok et al. (2023), none of these contrasts revealed univariate group differences. We therefore selected Large* > *Plus1 for decoding, as it maximized arithmetic-related signal while avoiding the dilution associated with the weaker Small* > *Plus1 contrast. For feature reduction, we constructed a union mask from the Large* > *Plus1 and Number* > *Shape (matching task) contrasts, comprising 24,669 voxels (17.54% of the whole brain; Supplementary Fig. S2). All voxels within the union mask served as input features for the EN classifier, which was trained to classify the DD versus TA groups using multivoxel data from the Large > Plus1 contrast values.

The significance of classification accuracy was determined through permutation tests with 5,000 iterations. The relative contribution of each voxel to classification (i.e., feature importance) was quantified using the coefficients estimated by the EN model. We first identified the peak coordinates of these brain feature clusters and created spherical regions of interest (ROIs) with a 6 mm radius centered on a peak coordinate. To further examine the directionality of group differences between the DD and TA groups, we calculated Cohen's *d* values within the identified ROIs. Brain regions were labeled by the Brainnetome atlas (Fan et al., 2016) and Julich atlas (Amunts et al., 2020). The full analysis pipeline is outlined in Figure 2.

**Figure 2.**
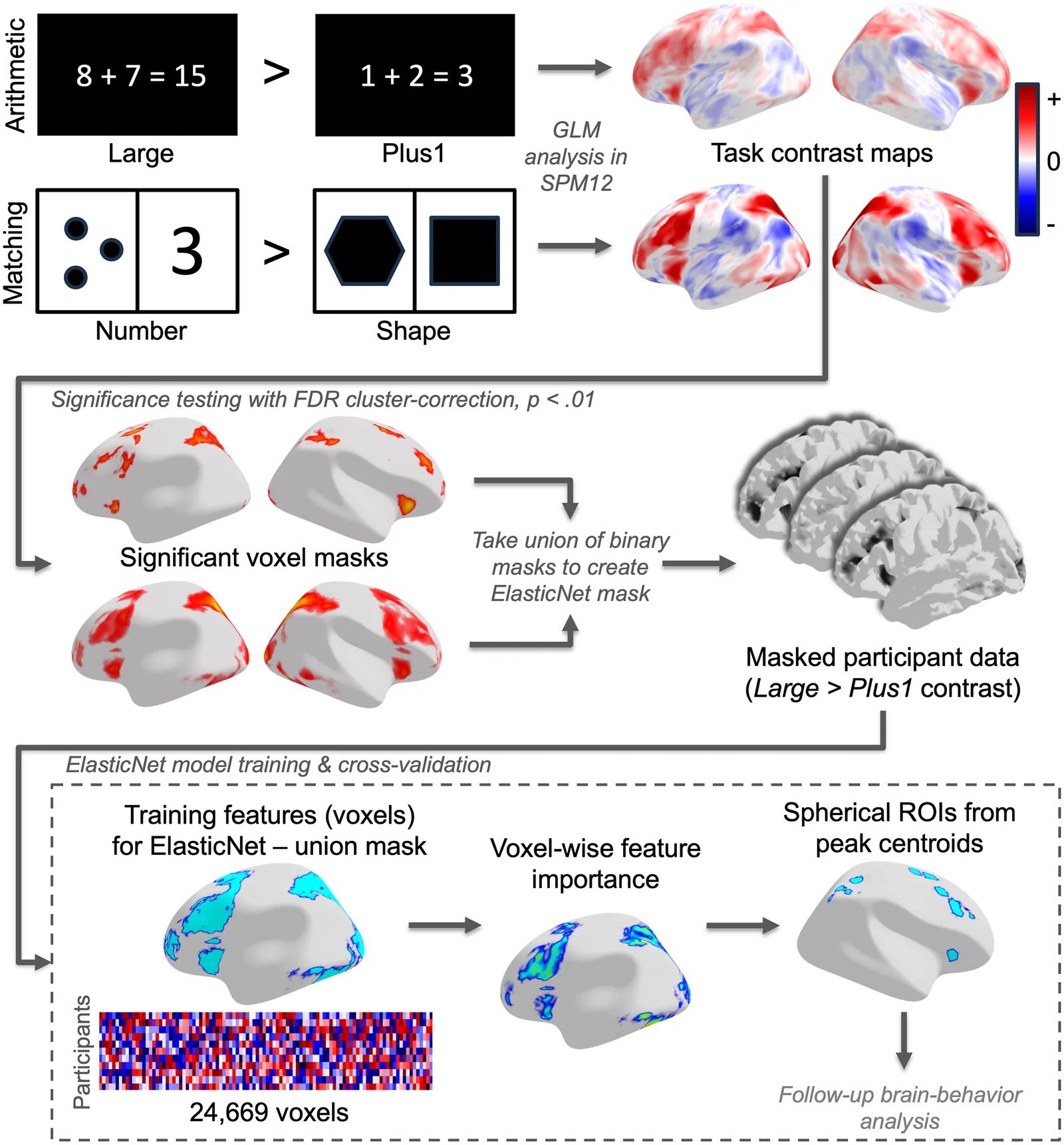
Analysis pipeline. GLM-based contrast generation. For the arithmetic verification task, participants determined whether addition equations were correct (e.g., 8 + 7 = 15) across three difficulty levels: Large problems (sum >10), Small problems (sum ≤10), and Plus1 problems (one addend equals 1). For the matching task, children judged whether paired stimuli matched across three conditions: Number (dots vs numerals), Shape (geometric forms), and Faces. We focused on the Large > Plus1 contrast for arithmetic and Number > Shape contrast for matching, as these produced the strongest within-subjects activation patterns (top panel). Mask creation and feature selection. Statistical significance testing with voxelwise FDR correction (*p* < 0.05) and cluster-correction (*p* < 0.01) identified significant voxels for each contrast, visualized on inflated brain surfaces (red/blue, positive/negative contrasts). Binary masks were created from these significant clusters and then combined via union operation to create a comprehensive mask for brain decoding (middle). Whole-brain Elastic Net decoding and ROI identification. Voxelwise activation values (Large* > *Plus1 contrast) served as input features for Elastic Net classification to distinguish DD from TA children. Model training employed LOOCV. Statistical significance was determined via permutation testing with 5,000 iterations (bottom).

#### Control analysis

Although analyzing correct trials only better captures neural signals of interest, the lower number of correct trials in the DD group compared with the TA group may have introduced a bias in the estimated group differences in neural response. To verify our results are not driven by this potential bias, we reran the second-level GLM and subsequent EN decoding analyses using contrasts derived from all trials. We then examined whether the brain feature weights identified by the original correct-trials-only EN model remain robust and consistent in the all-trials EN model. To this end, we performed a bivariate correlation of voxelwise coefficients estimated from each model within the brain mask defined by the original correct-trials-only EN model. Convergence between two models would confirm that the group differences identified by the correct-trials-only EN model are not driven by the lower trial count in the DD group.

#### Multivariate brain–behavior association analyses

To investigate whether functional brain features that distinguish children with DD from their TA peers are associated with latent cognitive mechanisms underlying arithmetic problem-solving, we conducted a multivariate canonical correlation analysis (CCA). This approach identifies linear combinations of brain measures (Large > Plus1 functional contrasts in the 38 ROIs identified by EN decoding, excluding 2 brainstem ROIs) and behavioral measures (BMAPS parameters of interest) that maximize the brain–behavior correlations. CCA yields a set of canonical modes, each consisting of a pair of linear combinations for the behavioral and brain datasets that maximize their correlation, along with loadings that indicate the contribution of each individual behavioral and brain measures to these modes. This analysis enables us to capture the shared variance between functional activity and latent cognitive processes involved in arithmetic problem-solving and to determine which ROIs/BMAPS parameters drive this relationship.

#### Transformer-based brain–behavior predictions

To further investigate which functional brain features predict specific latent cognitive mechanisms underlying arithmetic problem-solving, we employed Tabular Prior-data Fitted Network (TabPFN), a transformer-based foundation model designed for regression and classification on small- to medium-sized tabular datasets (Hollmann et al., 2025). Like large language models, TabPFN employs in-context learning, but rather than being trained on text, it is pretrained on synthetic tabular datasets generated from causal models and Bayesian neural networks. This allows to learn a broad prior over data-generating processes and autonomously acquire effective strategies for common real-world data challenges—such as missing values, outliers, and uninformative features—without requiring the hand-engineered preprocessing steps, required by classical regression or machine learning approaches. Critically, rather than returning a single point estimate for each observation as in classical regression, TabPFN approximates the posterior predictive distribution (PPD) for each new dataset, implementing prediction under Bayesian principles and providing uncertainty quantification alongside its predictions. This offers more statistically principled inference for small- to medium-sized datasets. TabPFN was chosen over traditional machine learning approaches as it can simultaneously adapt to both parametric and nonparametric structure without any hyperparameter tuning and has been shown to be competitive with, and in some cases outperform, LASSO regression even when parametric assumptions are correctly specified (Zhang et al., 2025).

Brain activity from each ROI was used as the feature vector input to TabPFN to predict individual BMAPS parameters that showed significant group differences between the DD and TA groups (see Results). Parameters of particular interest were the drift rates for each strategy, baseline switching time, switching sensitivity, and adaptivity. Model performance for each BMAPS parameter was assessed using *R*^2^ and Pearson's *r*, computed by correlating LOOCV-predicted values with observed BMAPS values. To confirm cross-model consistency, we additionally ran LASSO and ridge regression models and compared *R*^2^ and Pearson's *r* across all three approaches.

After confirming model consistency, feature importance for each ROI was computed using Shapley Additive Explanations (SHAP; Lundberg and Lee, 2017; Hollmann et al., 2025), a model-agnostic framework for interpreting predictions. For a given feature, SHAP values quantify its contribution to shifting the model's prediction away from the baseline, positively or negatively, for each individual datapoint. This yields both an average SHAP value per feature, reflecting its overall importance across the ROIs, and a distribution of individual SHAP values, one per participant.

### Code availability

All code used for this project will be shared via the Stanford Cognitive & Systems Neuroscience Laboratory GitHub webpage (https://github.com/scsnl).

## Results

### Poor arithmetic and number-matching performance in children with DD

We first examined whether children with DD exhibit differences in performance across conditions in the arithmetic verification task. Bayesian independent-samples *t* tests revealed that children with DD showed lower accuracy across conditions (BF_10_ = 126.53, δ = −0.90 [95% CIs = (−1.43, −0.39)]) compared with TA children, reproducing the findings of Kwok et al. (2023) (Table 2). No group differences were observed in total reaction times (RT; BF_10_ = 0.88, δ = 0.38 [95% CIs = (0.04, 0.86)]). Similar analysis conducted for each condition revealed group differences in the Large and Small conditions, but not in the Plus1 condition (BF_10_ = 0.53, δ = −0.28 [95% CIs = (−0.75, 0.17)]). In the Large condition, the DD group exhibited markedly lower accuracy than the TA group, yielding the largest observed group difference (BF_10_ = 705.81, δ = −1.05 [95% CIs = (−1.58, −0.51)]). No group difference was observed in RT (BF_10_ = 0.27, δ = −0.07 [95% CIs = (−0.52, 0.38)]). In the Small condition, the DD group also exhibited lower accuracy (BF_10_ = 35.04, δ = −0.79 [95% CIs = (−1.31, −0.29)]) and slower RT (BF_10_ = 10.52, δ = 0.67 [95% CIs = (0.19, 1.18)]) compared with the TA group.

**Table 2.** Mean accuracy and reaction times on arithmetic and matching tasks, and differences between the DD and TA groups

Condition	Accuracy (SD)		BF_10_	Reaction times (SD)		BF_10_
	TA	DD	Group differences	TA	DD	Group differences
Arithmetic verification task						
Total	87.3 (0.09)	76.4 (0.13)	126.53***	2,410 (430)	2,591 (410)	0.88
Large	79.2 (0.14)	61.5 (0.18)	705.81***	2,818 (574)	2,766 (685)	0.27
Small	89.1 (0.11)	77.4 (0.16)	35.04**	2,363 (505)	2,745 (507)	10.52**
Plus-1	93.8 (0.09)	90.5 (0.11)	0.53	2,049 (403)	2,264 (535)	1.06
Matching task						
Total	83.0 (0.06)	77.9 (0.08)	6.30*	1,404 (132)	1,431 (127)	0.34
Numbers	77.3 (0.10)	74.0 (0.09)	0.56	1,450 (160)	1,495 (146)	0.38
Shapes	93.0 (0.06)	89.0 (0.07)	1.99	1,241 (161)	1,299 (168)	0.61
Faces	79.0 (0.09)	71.2 (0.12)	10.52**	1,520 (151)	1,511 (194)	0.28

Mean reaction times and their standard deviations are rounded to the nearest millisecond.

* BF > 3, **BF > 10, ***BF > 10.

Similar group differences were also observed in the matching task. Children with DD showed lower accuracy (BF_10_ = 6.30, δ = −0.62 [95% CIs = (−1.12, −0.15)]) compared with TA children (Table 2), but no differences in RT (BF_10_ = 0.34, δ = 0.24 [95% CIs = (0.01, 0.66)]). Condition-specific analyses revealed a strong group difference in the Faces condition, with no effects in the Shapes (BF_10_ = 1.99, δ = −0.49 [95% CIs = (−0.98, −0.08)]) and Number condition (BF_10_ = 0.56, δ = −0.32 [95% CIs = (−0.78, −0.03)]). In the Faces condition, the DD group exhibited lower accuracy (BF_10_ = 10.34, δ = −0.67 [95% CIs = (−1.18, −0.19)]) than the TA group. None of the three conditions showed group differences in the RT (BF_10_ < 0.61). All group differences across conditions for both the arithmetic and matching tasks are visualized in Supplementary Figure S1.

These results suggest that group differences between the DD and TA groups, previously observed in Kwok et al. (2023), varied across task conditions.

### Lower efficiency in arithmetic strategy execution and selection in children with DD

Our first goal was to examine whether children with DD differ from their TA peers in arithmetic strategy selection and execution processes. The BMAPS (Mistry et al., 2025) estimated, for each trial, the probabilities of each strategy use (i.e., counting, retrieval, and decomposition), as well as cognitive parameters indexing strategy execution efficiency (e.g., strategy-specific drift rates) and metacognitive parameters related to strategy selection (e.g., baseline switching time and switching sensitivity).

To validate the BMAPS model in estimating strategy use, we performed a chi-squared test on the frequency of trials in which each strategy was used across small (sum ≤10), medium (sum = 11–12), and large (sum = 13–14) problem size bins. The results revealed a clear problem size effect: decomposition strategy use increases as the problem size increases (from 0.7% to 12.6% to 33.7% across small, medium, and large problems), whereas retrieval strategy use decreases as the problem size increases (from 55.5% to 4.4% and 7.1%). Results of the chi-squared test confirm that strategy dominance is dependent on problem size, χ^2^_(4)_ = 1,630.51, *p* < 0.001 (Supplementary Table S2).

Across trials, the averaged probability of each strategy was 9.6% (SD = 15.6%) for decomposition, 46.8% (SD = 40.4%) for retrieval, and 43.6% (SD = 35.6%) for counting. The proportion of trials in which each strategy had the highest probability of being selected was 7.6% for decomposition, 38.8% for retrieval, and 53.6% for counting. Trial-averaged strategy probabilities did not differ between TAs and DDs (*ps* > 0.694; Supplementary Table S1), suggesting that both groups relied on similar strategies overall, with decomposition being the least frequently used.

For the efficiency measures, independent-samples *t* tests revealed that children with DD exhibited lower efficiency for all three strategies—as indicated by reduced drift rates (retrieval: BF_10_ = 19.37; counting: BF_10_ = 348.10; decomposition: BF_10_ = 8.20 × 10^11^; Table 3, Fig. 3).

**Figure 3.**
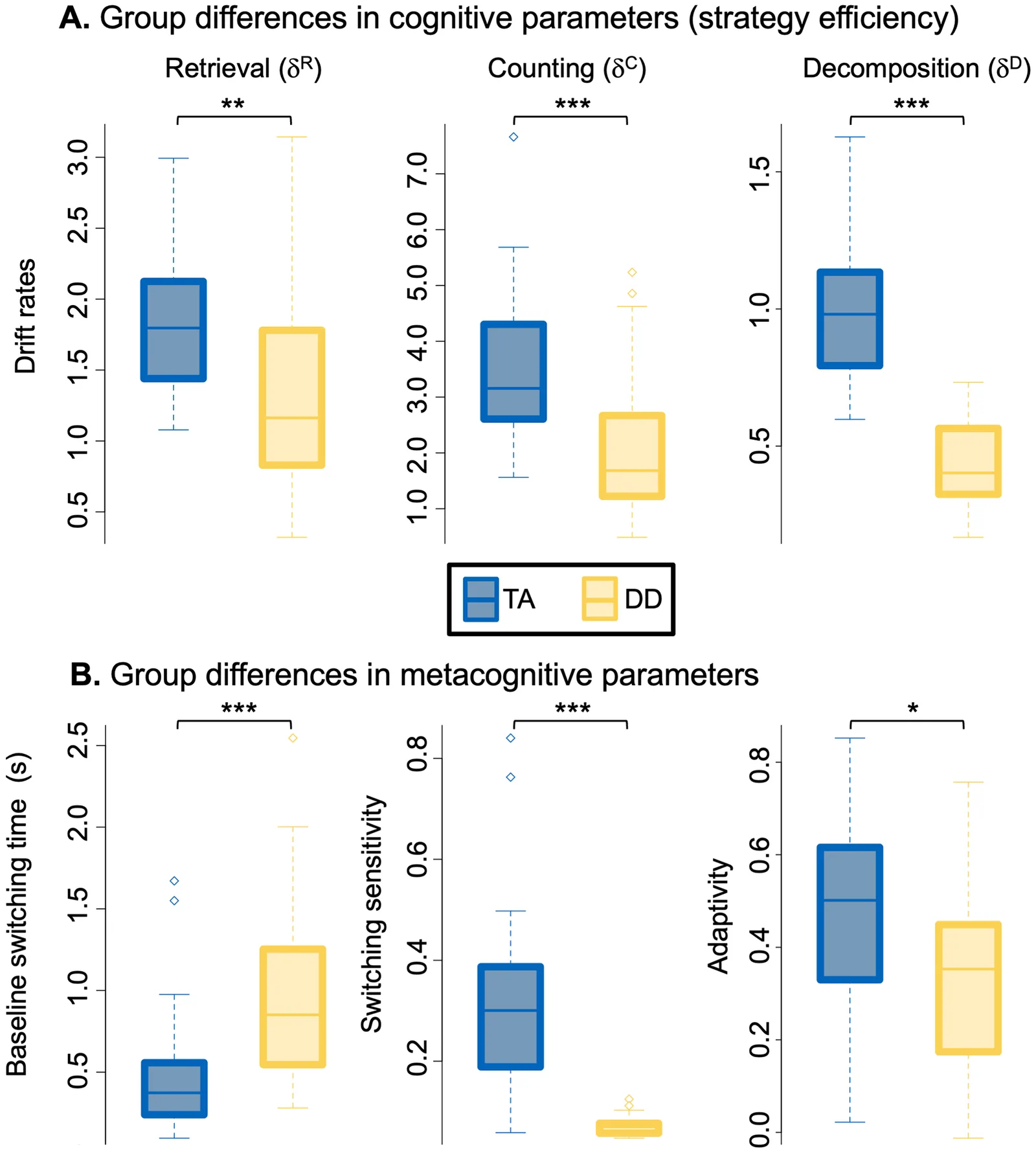
Children with DD exhibit deficits in both strategy execution efficiency and metacognitive control. ***A***, Cognitive parameters*.* Children with DD demonstrated substantially lower drift rates compared with TA children across all three strategies: Retrieval (*δ*^R^; Cohen's *d* = 0.834, BF₁₀ = 19.37), Counting (*δ*^C^; *d* = 1.098, BF₁₀ = 348.10), and Decomposition (*δ*^D^; *d* = 2.629, BF₁₀ = 8.20 × 10^11^). Lower drift rates indicate slower and less accurate accumulation of evidence toward correct responses. ***B***, Metacognitive parameters*.* Children with DD required more time to switch between strategies (Baseline switching time, γ; *d* = −1.088, BF₁₀ = 307.25), indicating reduced strategic flexibility. They were also less sensitive to problem difficulty when adjusting strategies (Switching sensitivity, ω; *d* = 1.829, BF₁₀ = 7.04 × 10^6^). Across trials, children with DD selected less optimal strategies (Adaptivity, Ψ; *d* = 0.666, BF₁₀ = 4.29). Together, these cognitive and metacognitive impairments indicate that children with DD have difficulties executing strategies efficiently and adaptively and flexibly switching between them. Yellow boxes, DD group; blue boxes, TA group. Box boundaries show the 25th–75th percentiles; center line, median. *p < 0.05, **p < 0.01, ***p < 0.001 based on Bayes factors. *d* indicates Cohen's *d.*

**Table 3.** Group differences in BMAPS parameters between the TA and DD groups and their correlations with in-scanner arithmetic performance

BMAPS parameter	Independent *t* tests (Cohen's *d*)	Pearson's *r*	
	TA > DD	Arithmetic acc.	Arithmetic RT
Retrieval drift rate	0.834**	0.844***	−0.261
Counting drift rate	1.098***	0.864***	−0.376**
Decomposition drift rate	2.629***	0.816***	−0.392**
Baseline switch time	−1.088***	−0.389**	0.626***
Switching sensitivity	1.829***	0.359*	0.059
Counting boost	0.797**	0.480***	−0.194
Adaptivity	0.666*	0.213	−0.289
Retrieval propensity	0.149	−0.230	−0.313*
Decision threshold	−0.484	−0.049	0.636***
Nondecision time	0.656*	0.463***	−0.117
L-R bias	0.237	0.243	−0.164
Mean entropy	0.253	−0.341*	−0.426**
Standard entropy	−0.088	0.266	0.294

Reported correlations between BMAPS parameters and accuracy/RT are presented for descriptive purposes only.

* BF_10_ > 3, **BF_10_ > 10, ***BF_10_ > 100.

Similar to the results with drift rates, children with DD showed deficits in metacognitive processes related to strategy selection, including longer baseline switching time (BF_10_ = 307.25), lower switching sensitivity (BF_10_ = 7.04 × 10^6^), and reduced adaptivity (BF_10_ = 4.29), compared with TA children.

These results indicate that although children with DD and TA children employed comparable strategies, both strategy execution efficiencies and metacognitive control involved in strategy selection may be impaired in children with DD.

### Whole-brain multivariate decoding distinguishes children with DD from typically achieving peers

Our second goal was to determine whether distributed functional brain patterns during arithmetic problem-solving jointly distinguish children with DD from their TA peers. We employed a whole-brain multivariate decoding approach using an Elastic Net (EN) classifier with cross-validation. The classifier assigned feature importance values to all voxels within a mask derived from the arithmetic verification task and matching tasks, indexing the relative contribution of each brain region to classifying the DD and TA groups (see details in Materials and Methods).

Decoding focused on the Large versus Plus1 contrast from the arithmetic verification task, which yielded the strongest and most widespread arithmetic-related activation and showed the most robust behavioral group difference of all conditions; the rationale for selecting this contrast, and for the 24,669-voxel union mask used for feature reduction, is detailed in Materials and Methods (Supplementary Tables S3, S4, Fig. S2). Consistent with Kwok et al. (2023), no univariate group differences were detected for any contrast.

The EN model successfully distinguished the DD group from the TA group, achieving a classification accuracy of 69.23%, (*p* = 0.028 by permutation testing). Table 4 reports model performance across EN mixing parameter *α* values). Results for alternative Large > Plus1 and Number > Shape mask models are provided in Supplementary Table S5.

**Table 4.** Elastic Net (EN) neural decoding performance depending on alpha value

*α*	Accuracy	Sensitivity	Specificity	TN	FP	FN	TP	*p* value
0	0.5692	0.4286	0.6757	25	12	16	12	0.225
0.25	0.6000	0.2143	0.8919	33	4	22	6	0.163
0.5	0.6154	0.3929	0.7838	29	8	17	11	0.131
0.75	0.6154	0.2857	0.8649	32	5	20	8	0.112
0.9	0.6462	0.3571	0.8649	32	5	18	10	0.044*
1	0.6923	0.4643	0.8649	32	5	15	13	0.028*

Larger *α* values incur a greater penalty for having many nonzero features. TN, true negative (TA correctly categorized as TA); FP, false positive, FN, false negative; TP¸ true positive (DD correctly categorized as DD). Sensitivity = TP / (TP + FN). Specificity = TN / (TN + FP). *p* values are based on permutation testing of model accuracy with 5,000 iterations.

* *p* < 0.05.

To identify regions contributing most to classification, we extracted voxels with peak feature importance from all significant clusters and defined 6 mm spherical ROIs centered on these peaks. The resulting 40 ROIs spanned distributed cortical and subcortical regions, including the bilateral inferior and superior parietal lobules (IPL/SPL) including the intraparietal sulcus (IPS), superior and middle frontal gyrus (SFG/MFG), precentral gyrus (PreCG), lingual gyrus (LG), and fusiform gyrus (FG), as well as the right hippocampus and thalamus (Fig. 4).

**Figure 4.**
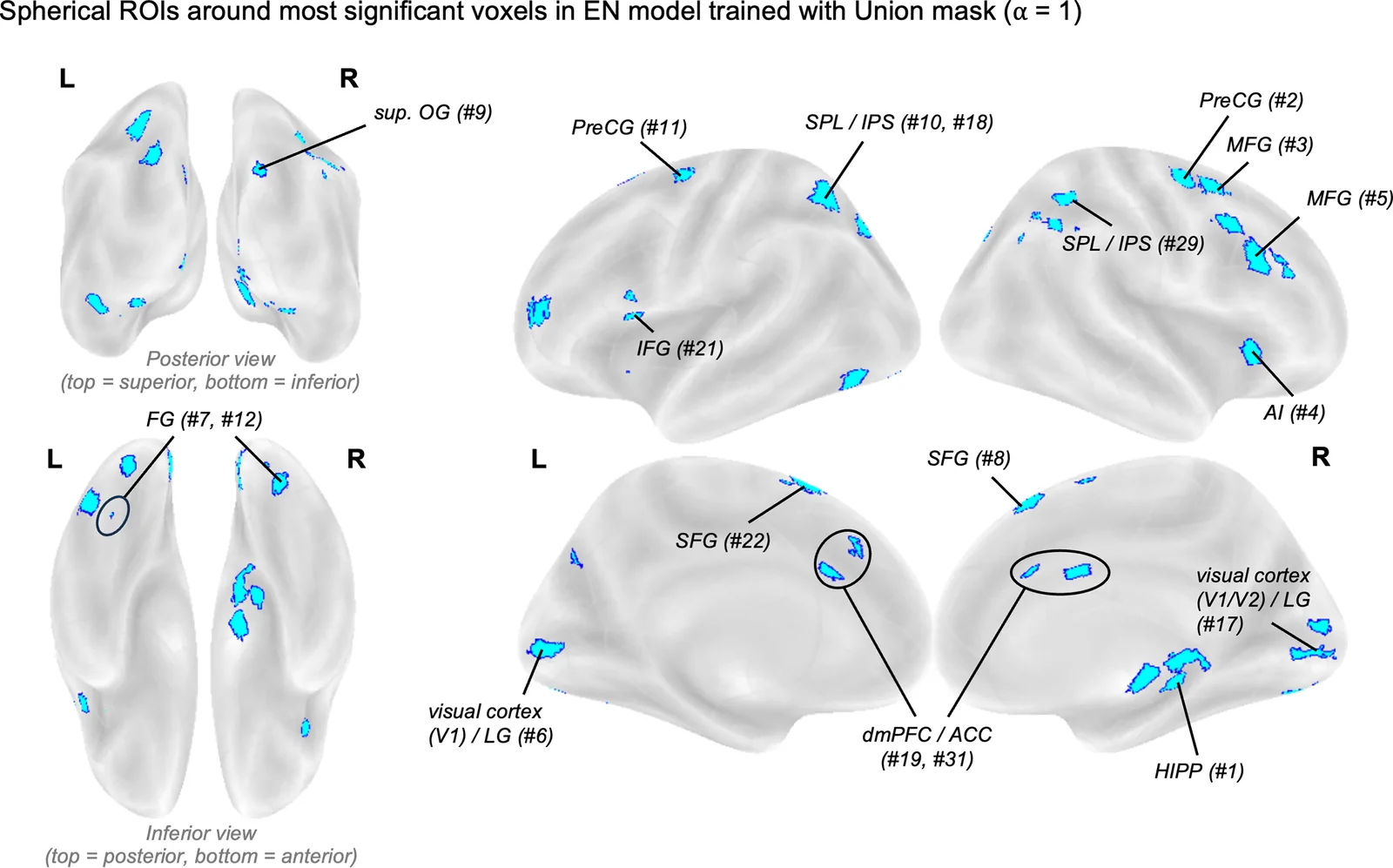
Distributed neural signature of developmental dyscalculia (DD) identified through whole-brain Elastic Net decoding encompasses frontoparietal, hippocampal, and visual regions. Whole-brain Elastic Net classification with cross-validation identified brain regions whose distributed activation patterns jointly distinguished children with DD from TA peers with (*p* = 0.028, permutation testing). Brain regions are mapped to the Brainnetome atlas (Fan et al., 2016). Key regions included the bilateral precentral gyrus, right middle frontal gyrus, superior occipital gyrus, right hippocampus, right superior/middle/inferior frontal gyrus, bilateral superior and inferior parietal lobules including intraparietal sulcus, fusiform gyrus, and areas of the visual cortex including the lingual gyrus. Numbered labels for each cluster from Table 5 are shown in brackets. Abbreviations (ROIs): HIPP, hippocampus; PreCG, precentral gyrus; MFG, middle frontal gyrus; AI, anterior insula; LG, lingual gyrus; FG, fusiform gyrus; SFG, superior frontal gyrus; OG, occipital gyrus; SPL, superior parietal lobule; IPS, intraparietal sulcus; dmPFC, dorsomedial prefrontal cortex; ACC, anterior cingulate cortex; IFG, inferior frontal gyrus; sup., superior.

We then quantified the direction and magnitude of group differences in each ROI using Cohen's *d* (Table 5). The TA group showed greater activation in 26 of the 40 ROIs, while the DD group showed greater activation in the remaining 14. Among regions with the highest EN decoding coefficients, those with greater activation in the TA group included the right hippocampus (HIPP); right SFG, MFG, inferior frontal gyrus (IFG); right LG, right FG; and left SPL/IPL including the IPS. Regions with the highest peak coefficients that had higher activation in DDs included the bilateral PreCG and right MFG.

**Table 5.** Brain regions identified by Elastic Net (EN) neural decoding with different features between the DD and TA group in the arithmetic verification task

Centroid #	Region	*x*	*y*	*z*	Peak coefficient	Cohen's *d*
DD > TA						
2	R PreCG	26	−10	50	1.8220	−0.556
3	R MFG	26	4	48	1.7315	−0.505
9	R sup. OG	26	−78	40	0.5866	−0.337
11	L PreCG	−24	−12	52	0.5191	−0.348
13	L occipital FG	−40	−66	−10	0.4576	−0.020
19	L dmPFC/ACC	−2	24	34	0.3208	−0.313
20	L sup. OG	−20	−68	36	0.3126	−0.215
21	L IFG	−46	16	0	0.3085	−0.067
22	L SFG	−14	16	66	0.2867	−0.442
23	Medial SFG	0	2	72	0.2839	−0.177
24	L frontal pole	−40	52	14	0.2505	−0.064
27	L SFG	−10	12	66	0.2326	−0.442
33	L SFG	−10	14	64	0.0712	−0.313
36	L frontal pole	−36	48	12	0.0379	−0.113
TA > DD						
1	R HIPP	32	−32	0	2.3527	0.714
4	R AI	28	28	2	1.4670	0.653
5	R MFG	40	20	30	0.9132	0.637
6	L visual cortex (V1)/LG	−14	−86	2	0.8723	0.768
7	L FG	−36	−64	−12	0.6797	0.002
8	R SFG	6	24	60	0.6692	0.334
10	L SPL/IPS	−28	−56	56	0.5764	0.317
12	R occipital FG	30	−80	−6	0.4622	0.836
14	R MFG	42	12	42	0.4464	0.670
15	R brainstem	10	−22	−12	0.4218	0.517
16	L occipital FG	−26	−84	−18	0.3785	0.723
17	R visual cortex (V1/V2)/LG	12	−86	−2	0.3639	0.889
18	L SPL/IPS	−28	−56	52	0.3239	0.459
25	L IFG	−46	14	16	0.2376	0.627
26	R IPS/AG	46	−48	46	0.2374	0.770
28	R thalamus	16	−32	2	0.2159	0.665
29	R SPL/IPS	36	−46	48	0.1878	0.453
30	R Visual Cortex (V1/V2)/LG	20	−86	−2	0.1537	0.808
31	R ACC	8	4	28	0.1359	0.551
32	R V1/LG	4	−80	6	0.1109	0.531
34	R MFG	52	36	28	0.0577	0.653
35	R brainstem	10	−22	−8	0.0398	0.412
37	R MFG	48	32	38	0.0321	0.734
38	R LOC/IPS	40	−60	40	0.0320	0.723
39	R LOC/IPS	38	−60	38	0.0159	0.723
40	R thalamus	22	−30	4	0.0049	0.631

Mean peak coefficient is 0.465 (SD = 0.527). Cohen's *d* indicates the effect size associated with a *t* test comparing functional activity between the TAs and DDs for the Large* > *Plus1 contrast. Abbreviations (ROIs): HIPP, hippocampus; PreCG, precentral gyrus; MFG, middle frontal gyrus; AI, anterior insula; LG, lingual gyrus; FG, fusiform gyrus; SFG, superior frontal gyrus; OG, occipital gyrus; SPL, superior parietal lobule; IPS, intraparietal sulcus; dmPFC, dorsomedial prefrontal cortex; ACC, anterior cingulate cortex; IFG, inferior frontal gyrus; AG, angular gyrus; LOC, lateral occipital cortex; sup., superior.

Together, these results show that children with DD exhibit functional activation patterns that are reliably distinguishable from those of their TA peers across distributed brain regions, even in the absence of detectable univariate group differences.

### Control analysis: decoding is robust to trial-count differences between groups

Because only correct trials were included in the primary decoding analysis, and children with DD answered fewer trials correctly than TA children, the lower trial count in the DD group could in principle have biased the estimated group differences. To evaluate this possibility, we reran the EN decoding analysis using Large* > *Plus1 contrast maps generated from all trials, regardless of accuracy, thereby equating the two groups more closely in the number of trials contributing to each contrast map. We then correlated the voxelwise EN coefficients from this all-trials model with those from the original correct-trials-only model, restricted to the brain mask defined by the original model.

The two sets of coefficients were strongly and significantly correlated (*r* = 0.68, *p* < 0.001; Supplementary Fig. S3), indicating that the spatial pattern of regional contributions to classification was largely preserved when all trials were included. This convergence confirms that the group differences identified by our primary decoding model are robust to differences in trial count between groups and are unlikely to be artifacts of the lower number of correct trials in the DD group.

### Distributed brain activity patterns predict latent cognitive and metacognitive processes: canonical correlation analysis

Having shown that distributed activation patterns distinguish the DD and TA groups, we next asked whether and how these patterns relate to the latent cognitive and metacognitive processes underlying arithmetic problem-solving. To capture system-level brain–behavior associations across the DD and TA groups, we conducted a canonical correlation analysis (CCA) relating the full set of EN-derived ROIs (brain data) to five BMAPS parameters (behavioral data): counting and retrieval drift rates, switching sensitivity, baseline switching time, and adaptivity. Decomposition drift rate was excluded because the probability of the decomposition strategy was rarely selected (<10% of trials in both groups) and carried high error rates (∼50%), making it unlikely that activation would reliably track its use.

This analysis revealed a single significant canonical mode with a brain–behavior correlation of *r* = 0.956 (*R*^2^ = 0.243), which distinguished the DD and TA groups along both the behavioral and brain dimensions (Fig. 5*A*). Among behavioral parameters, counting drift rate and switching sensitivity loaded most strongly, whereas baseline switching time loaded most strongly in the negative direction (Fig. 5*B*, Table 6). Among brain regions, the right HIPP, left SPL/IPS, right LG, and right cuneus showed the highest positive loadings, and the left SFG the highest negative loadings (Fig. 5*C*, Table 6, Supplementary Fig. S4).

**Figure 5.**
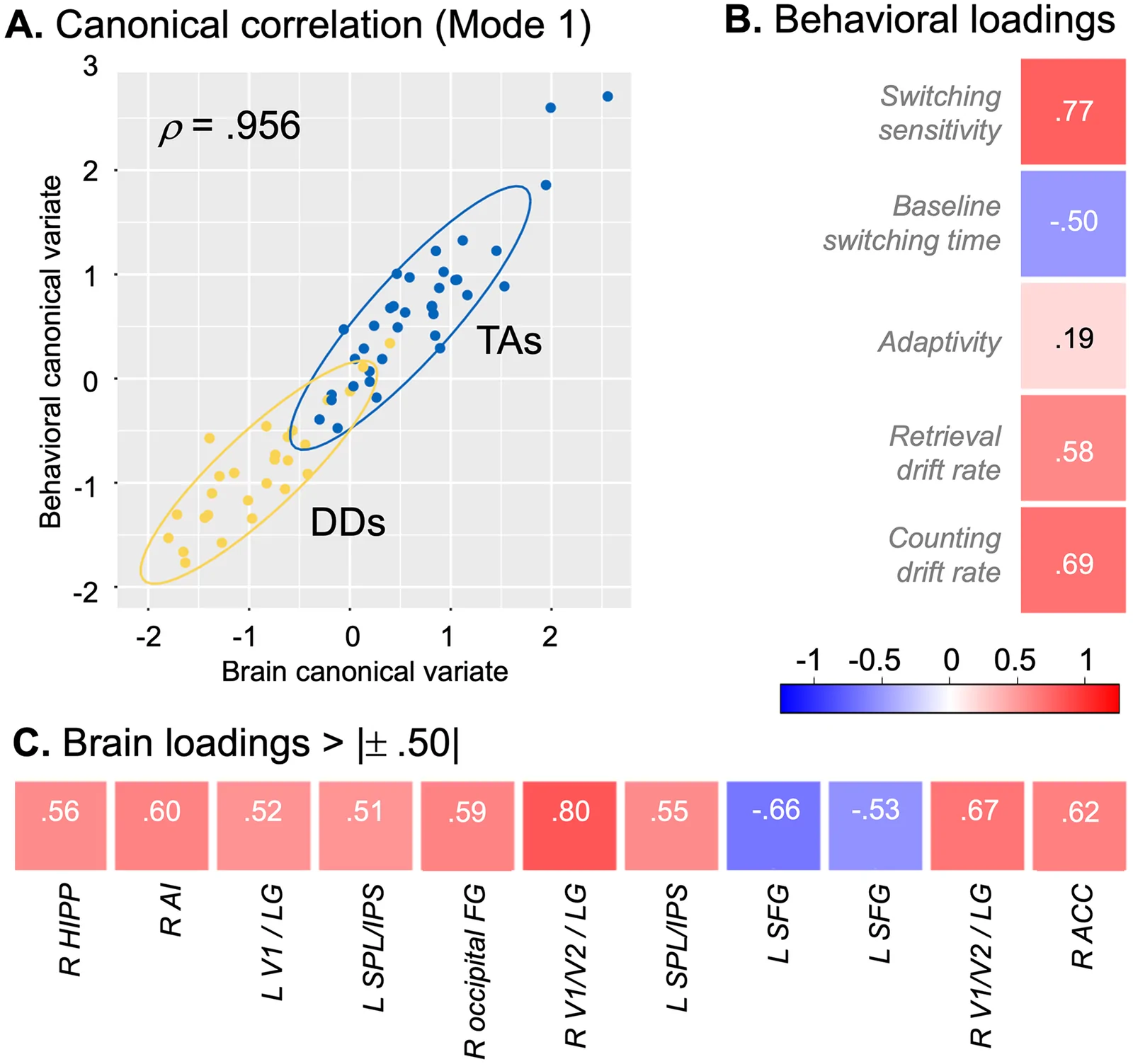
Canonical correlation analysis revealed system-level brain–behavior associations across the DD and TA groups. ***A***, First canonical mode maximizing the brain–behavior correlation. Scatter plot shows the relationship between *z*-scores of the first canonical variate's brain measures (*x*-axis) and behavioral measures (*y*-axis). Brain data were derived from 38 cortical-subcortical ROIs and behavioral data were derived from five BMAPS parameters. The first canonical mode achieved a strong brain–behavior correlation of 0.956 (*R*^2^ = 0.243, *p* < 0.001), effectively distinguishing the DD group (yellow points/ellipse) from the TA group (blue points/ellipse) across both neural and behavioral dimensions. Each point represents individual participant. Ellipses indicate 90% confidence regions. This system-level covariation demonstrates that distributed neural patterns and latent cognitive/metacognitive processes jointly characterize DD status. ***B***, Behavioral loadings. Positive loadings were found in switching sensitivity (ω; 0.77), counting drift rate (δ^C^; 0.69), retrieval drift rate (δ^R^; 0.58), and adaptivity (Ψ; 0.19). Negative loadings were found in baseline switching time (γ; −0.50). ***C***, Brain loadings. Strongest positive loadings were observed in right visual regions and the lingual gyrus (LG; 0.80 and 0.67), right anterior cingulate gyrus (ACC; 0.62), right anterior insula (AI; 0.60), right fusiform gyrus (FG; 0.59), right hippocampus (HIPP; 0.56), and two clusters in the left superior parietal lobule/intraparietal sulcus (SPL/IPS; 0.55 and 0.51). Negative loadings were observed in two clusters in the left superior frontal gyrus (SFG; −0.66 and −0.53). Only loadings with absolute values exceeding 0.50 are displayed; complete loadings can be found in Table 6. Loadings for all canonical modes are illustrated in Supplementary Figure S4. Abbreviations: DD, developmental dyscalculia; TA¸ typically achieving.

**Table 6.** Canonical loadings for brain/behavioral variables onto first canonical mode (CCA analysis)

Behavioral loadings		Brain loadings	
Positive loadings			
Switching sensitivity	0.77	R visual cortex (V1/V2)/LG (17)	0.80
Counting drift rate	0.69	R visual cortex (V1/V2)/LG (30)	0.67
Retrieval drift rate	0.58	R ACC (31)	0.62
Adaptivity	0.19	R AI (4)	0.60
		R occipital FG (12)	0.59
		R HIPP (1)	0.56
		L SPL/IPS (18)	0.55
		L visual cortex (V1)/LG (6)	0.52
		L SPL/IPS (10)	0.51
Negative loadings			
Baseline switching time	−0.50	L SFG (22)	−0.66
		L SFG (27)	−0.53

Cluster numbers are given in parentheses besides the name of each ROI. To save space, only ROIs with loadings greater than |±0.50| are shown.

Abbreviations (ROIs): HIPP, hippocampus; AI, anterior insula; LG, lingual gyrus; FG, fusiform gyrus; SFG, superior frontal gyrus; SPL, superior parietal lobule; IPS, intraparietal sulcus; ACC, anterior cingulate cortex.

Together, these results provide system-level evidence that the distributed neural signature distinguishing DD from TA children is jointly organized with the latent cognitive and metacognitive parameters governing arithmetic problem-solving, establishing a brain–behavior axis that we next decompose at the level of individual processes.

### Distributed brain activity patterns predict latent cognitive and metacognitive processes: TabPFN prediction analysis

To identify which neural regions support each latent process and whether their contributions differ between groups, we used a transformer-based foundation model approach, the TabPFN (Hollmann et al., 2025), to predict BMAPS parameters from ROI activation, and quantified each region's contribution using normalized SHAP values. We then compared mean SHAP values between TA and DD children for each ROI (Table 7). Significant LOOCV results emerged for counting drift rate (*R*^2^ = 0.053, Pearson's *r* = 0.271, *p* = 0.032), decomposition drift rate (*R*^2^ = 0.205, *r* = 0.467, *p* < 0.001), and switching sensitivity (*R*^2^ = 0.189, *r* = 0.492, *p* < 0.001). To evaluate cross-method consistency, we additionally ran LASSO and ridge regression models. Results from both methods were consistent with TabPFN, yielding significant prediction to counting drift rates, decomposition drift rate, and switching sensitivity, and nonsignificant predictions for the remaining parameters (see details in Supplementary Table S6). These results confirm the robustness of our predictions.

**Table 7.** Group differences in mean normalized SHAP values between TAs and DDs for each ROI in the TabPFN models for switching sensitivity (*ω*) and counting/decomposition drift rates (*δ*^C^, *δ*^D^)

	Region	SHAP						*p* value		
		*ω*		*δ* ^C^		*δ* ^D^				
		TA	DD	TA	DD	TA	DD	*ω*	*δ* ^C^	*δ* ^D^
1	R HIPP	0.051	−0.031	0.126	−0.220	0.088	−0.193	<0.001**	0.008*	0.001*
2	R PreCG	0.144	−0.047	0.020	0	0.044	−0.015	<0.001**	0.098	0.001*
3	R MFG	0.071	−0.026	0.006	0.001	0.026	−0.016	<0.001**	0.581	0.005*
4	R AI	−0.084	−0.072	0.008	−0.003	0.013	−0.012	0.010	0.332	0.001*
5	R MFG	0.024	−0.019	0.033	0.006	0.027	−0.011	<0.001**	0.025	0.001*
6	L V1 and LG	−0.017	−0.021	0.013	−0.061	0.054	−0.066	<0.001**	0.001*	<0.001**
7	L FG	0.02	−0.025	−0.003	−0.015	0.015	−0.012	<0.001**	0.175	0.007*
8	R SFG	−0.008	−0.013	0.019	−0.019	0.019	−0.044	<0.001**	0.027	0.002*
9	R sup. OG	0.047	−0.038	0.009	−0.04	0.041	−0.013	0.005*	0.001*	0.003*
10	L SPL/IPS	−0.047	−0.023	0.009	−0.024	0.011	−0.004	0.005*	0.114	0.036
11	L PreCG	−0.008	−0.011	−0.011	−0.011	0.023	−0.003	<0.001**	0.898	0.002*
12	R occipital FG	−0.016	−0.044	−0.007	0.005	0.017	−0.018	<0.001**	0.533	<0.001**
13	L occipital FG	0.04	−0.024	0.039	0.012	0.022	−0.009	0.006*	0.166	0.054
14	R MFG	0.042	−0.018	0.027	−0.018	0.014	−0.031	0.001*	0.005*	0.005*
16	L occipital FG	0.003	−0.011	0.014	−0.013	0.011	−0.035	<0.001**	0.139	0.019
17	R V1/V2 and LG	0.007	−0.023	0.078	−0.128	0.077	−0.141	<0.001**	<0.001**	<0.001**
18	L SPL/IPS	−0.012	−0.035	0.008	−0.012	−0.001	−0.024	0.003*	0.056	0.008*
19	L dmPFC/ACC	0.034	−0.012	0.051	−0.019	0.034	−0.013	<0.001**	0.001*	<0.001**
20	L sup. OG	0.048	−0.018	0.014	0	0.012	0.004	<0.001**	0.142	0.245
21	L IFG	0.025	−0.049	−0.003	−0.018	0.014	−0.012	0.009*	0.412	0.002*
22	L SFG	−0.017	−0.016	0.133	−0.118	0.115	−0.093	<0.001**	0.005*	0.001*
23	Medial SFG	0.016	−0.009	0.024	−0.009	0.026	−0.020	<0.001**	0.119	0.002*
24	L frontal pole	0.001	−0.005	0.005	0.003	0.011	0.003	<0.001**	0.931	0.242
25	L IFG	0.008	−0.049	0.043	−0.017	0.017	−0.020	<0.001**	0.001*	<0.001**
26	R IPS/AG	−0.015	−0.014	−0.010	−0.01	0.015	0.001	0.138	0.872	0.048
27	L SFG	0.002	−0.010	0.041	−0.025	0.064	−0.030	<0.001**	0.002*	<0.001**
28	R thalamus	−0.017	−0.021	0.027	−0.003	0.010	−0.005	<0.001**	0.020	0.039
29	R SPL/IPS	−0.022	−0.073	0.010	−0.040	0.001	−0.019	0.004*	0.009*	0.003*
30	R V1/V2 and LG	0.036	−0.084	−0.009	−0.025	0.012	−0.032	<0.001**	0.082	<0.001**
31	R ACC	−0.013	−0.015	0.030	−0.029	0.023	−0.025	<0.001**	0.047	<0.001**
32	R V1/LG	0.003	−0.012	0.034	−0.020	0.026	−0.015	<0.001**	<0.001**	<0.001**
33	L SFG	0.005	−0.010	0.024	−0.018	0.032	−0.007	<0.001**	0.004*	<0.001**
34	R MFG	−0.001	−0.019	0.012	0.020	0.004	−0.014	<0.001**	0.334	0.058
36	L frontal pole	−0.011	−0.008	0.034	0.004	0.018	−0.001	<0.001**	0.031	0.034
37	R MFG	−0.016	−0.029	−0.008	−0.003	−0.002	−0.002	<0.001**	0.670	0.985
38	R LOC/IPS	−0.019	−0.010	0.007	0.001	0.008	−0.005	0.001*	0.620	0.070
39	R LOC/IPS	−0.009	−0.016	0.005	0.020	0.009	−0.007	<0.001**	0.392	0.035
40	R thalamus	−0.012	−0.021	0.026	0.005	0.023	−0.010	<0.001**	0.058	<0.001**

Abbreviations (ROIs): HIPP, hippocampus; PreCG, precentral gyrus; MFG, middle frontal gyrus; AI, anterior insula; LG, lingual gyrus; FG, fusiform gyrus; SFG, superior frontal gyrus; OG, occipital gyrus; SPL, superior parietal lobule; IPS, intraparietal sulcus; dmPFC, dorsomedial prefrontal cortex; ACC, anterior cingulate cortex; IFG, inferior frontal gyrus; AG, angular gyrus; LOC, lateral occipital cortex; sup., superior.

* *p* < 0.01, **p < 0.001. Significance determined using an α level of 0.01. Brainstem clusters not shown.

### Distributed brain activity patterns predict latent cognitive and metacognitive processes: TabPFN-based feature extraction

Across all three parameters, group comparisons revealed a striking and consistent reversal in the directionality of regional contributions: SHAP values were positive or near-zero in TA children but negative in DD children, such that contributions were systematically higher in TA than DD children (Table 7, Fig. 6, Supplementary Fig. S5). Thus, the same distributed regions that contributed positively to predicting these behavioral parameters in TA children contributed negatively in children with DD.

**Figure 6.**
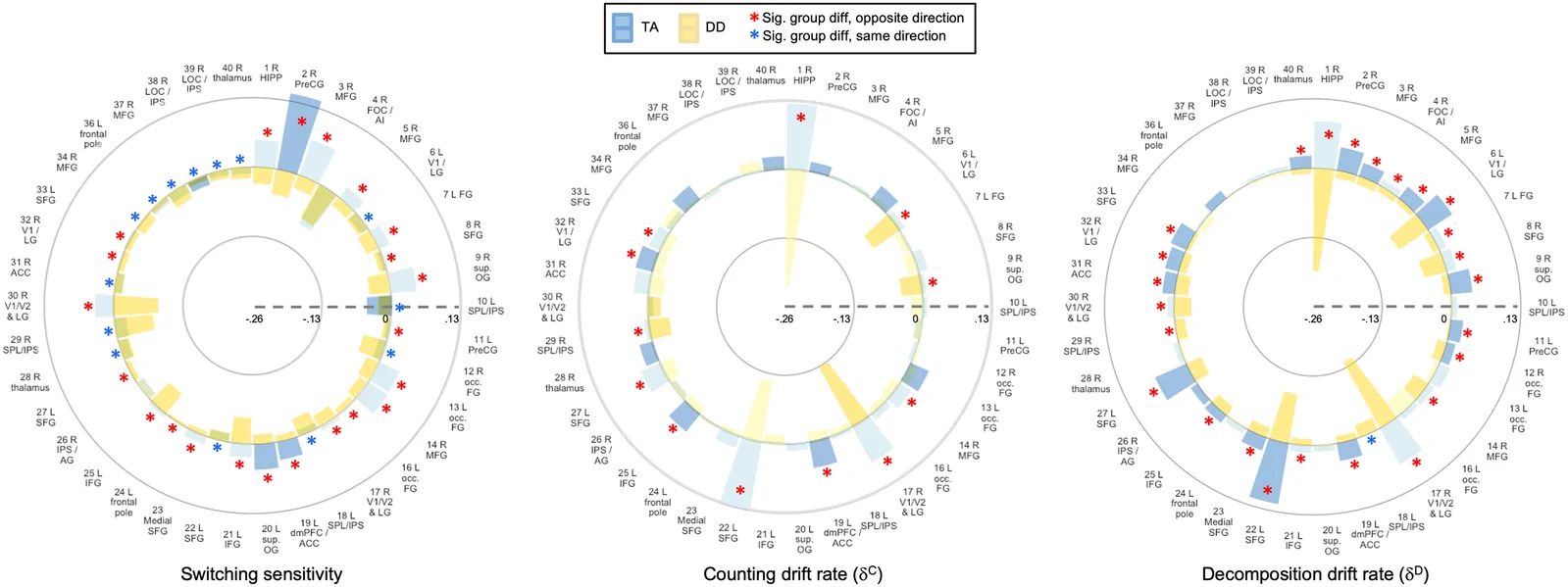
Distributed brain activity patterns predict metacognitive switching sensitivity and procedural strategy efficiency, with the directionality of regional contributions differing across DD and TA groups. TabPFN feature importance was estimated using Shapley Additive Explanations (SHAP); values indicate the magnitude and direction of each ROI's contribution across participants for each BMAPS parameter. SHAP values reveal a major divergence between the TA and DD groups in both the magnitude and direction of regional contributions. The most pronounced group reversals were found in the right hippocampus for all three BMAPS parameters (positive in the TA group, negative in the DD group), as well as in the dmPFC/ACC and visual cortex. Group differences were most pervasive for switching sensitivity (36 ROIs; brainstem ROIs not shown), with 22 ROIs showing strong group reversals, especially in the right PreCG, right MFG, and right visual cortex. For strategy efficiency, both drift rates showed more focal group differences, though most were in opposite directions between groups. Counting drift rates were concentrated in the left SFG, bilateral visual cortex, and right SPL/IPS. Decomposition drift rates showed group reversals across more widespread brain regions compared with counting, including the frontal and fusiform gyrus. Regions labeled with an asterisk (*) show a statistically significant (*p* < 0.01) divergence for feature importance between TAs and DDs, while label color indicates whether the mean SHAP values for TAs and DDs have the same (e.g., positive SHAP in both groups with group difference in magnitude only) or opposite (e.g., positive SHAP in TAs vs negative SHAP in DDs) sign: red, opposite; blue, same. Bar shading (light vs dark) indicates statistical significance of SHAP values based on within-group one-sample *t* tests for that region (light, not significant; dark, significant). Abbreviations (ROIs): HIPP, hippocampus; PreCG, precentral gyrus; MFG, middle frontal gyrus; AI, anterior insula; LG, lingual gyrus; FG, fusiform gyrus; SFG, superior frontal gyrus; OG, occipital gyrus; SPL, superior parietal lobule; IPS, intraparietal sulcus; dmPFC, dorsomedial prefrontal cortex; ACC, anterior cingulate cortex; IFG, inferior frontal gyrus; AG, angular gyrus; LOC, lateral occipital cortex; sup., superior.

This directional reversal was most pervasive for switching sensitivity, our index of metacognitive control, where 36 of 38 ROIs (not including two brainstem clusters) differed significantly between groups (most at *p* < 0.001) with 22 ROIs showing group reversals, spanning frontal, parietal, hippocampal, visual, and subcortical regions. This near brain-wide group difference indicates that metacognitive control over strategy selection is supported by broadly positive distributed contributions in TA children but broadly negative contributions in DD. Group differences were also widespread for decomposition drift rate (26 of 38 ROIs) and more focal for counting drift rate (12 of 38 ROIs): both showed significant differences across distributed hippocampal, frontal, parietal, and dorsomedial frontal regions, while the latter was limited to hippocampus, lingual gyrus, superior frontal gyrus, and visual cortex. In every case, the significant differences followed the same TA-positive, DD-negative pattern.

The right hippocampus showed the most pronounced group reversal across all three parameters. Its contribution to counting drift rate reversed from positive in TA children (mean SHAP = 0.126) to strongly negative in DD children (−0.220; *p* = 0.008), with parallel reversals for decomposition drift rate (0.088 vs −0.193; *p* = 0.001) and switching sensitivity (0.051 vs −0.031; *p* < 0.001). Comparable counting-related reversals were observed in the right visual cortex/lingual gyrus (clusters 17 and 32: 0.078 vs −0.128, *p* < 0.001; and 0.034 vs −0.020; *p* < 0.001) and left superior frontal gyrus (clusters 22, 27, and 33: 0.133 vs −0.118, *p* = 0.005; 0.041 vs −0.025, *p* = 0.002; and 0.024 vs −0.018, *p* = 0.004; Table 7).

Together, these findings indicate that the distributed neural architecture supporting arithmetic strategy execution and metacognitive control is engaged in opposing directions in children with DD relative to their TA peers, with hippocampal disengagement during procedural computation as the most prominent group-distinguishing feature.

## Discussion

We investigated cognitive, metacognitive, and neural mechanisms underlying arithmetic problem-solving deficits in children with developmental dyscalculia (DD), integrating Bayesian computational modeling of behavior with multivariate brain-wide decoding of fMRI data from Kwok et al. (2023). Three key insights emerged: first, children with DD exhibit deficits in both strategy execution efficiency, indexed by reduced drift rates for counting, retrieval, and decomposition, and metacognitive control, indexed by impaired switching sensitivity, longer switching times, and reduced adaptivity. Second, whole-brain Elastic Net classification identified distributed neural patterns across frontoparietal, hippocampal, and visual regions that distinguished DD from typically achieving (TA) children. Third, canonical correlation analysis (CCA) and transformer-based predictions converged in showing that these distributed neural signatures predicted latent cognitive and metacognitive parameters, with the intraparietal sulcus (IPS), hippocampus, and frontoparietal cortical regions as critical nodes. Together, our findings provide the first comprehensive, mechanistic account linking latent cognitive processing deficits to their neural substrates during arithmetic problem-solving in DD.

Our application of the BMAPS (Supekar et al., 2021; Mistry et al., 2025; Fig. 1) revealed pervasive deficits in DD extending beyond accuracy or strategy preferences. Both groups relied primarily on counting and retrieval strategies, with minimal use of decomposition, consistent with typical development at ages 8–10 for the problem set used in our study (Geary et al., 2004). Critically, no group differences emerged in the proportion of chosen strategies, confirming that children with DD employ the same strategic repertoire as their TA peers. However, they executed all three strategies less efficiently (Fig. 3*A*), with lower drift rates for counting (*d* = 1.098), retrieval (*d* = 0.834), and decomposition (*d* = 2.629), indicating less accurate evidence accumulation. These results extend prior reports showing reduced accuracy in children with mathematical learning disabilities (Russell and Ginsburg, 1984; Geary, 1990; Geary et al., 2012) by quantifying trial-by-trial execution efficiency through rigorous computational modeling.

Children with DD exhibited pronounced impairments in metacognitive control (Fig. 3*B*). Longer baseline switching time (*d* = −1.088) reflect general delays in switching from the initially chosen strategy, while reduced switching sensitivity (*d* = 1.829) revealed that their decisions to switch strategies were insensitive to problem difficulty. Unlike TA children, they did not modulate switching according to whether problems were easy or difficult. Reduced adaptivity (*d* = 0.666) further indicated that when they did switch strategies, their choices were often suboptimal. This pattern suggests that children with DD may lack both awareness that their current strategy is failing and the flexibility to shift adaptively to more effective approaches, leading to persistent use of suboptimal strategies. Because metacognition encompasses not only control but also monitoring processes such as error detection and performance evaluation (Nelson and Narens, 1990), and because effective switching presupposes accurate monitoring, future studies should adopt experimental and analytic designs that dissociate monitoring from control to clarify how the two interact in DD.

Our whole-brain decoding with Elastic Net revealed distributed brain activation patterns jointly distinguishing the groups (Fig. 4; Tables 4, 5), a result particularly notable given that univariate analyses of these data yielded null results (Kwok et al., 2023), underscoring the importance of multivariate approaches for characterizing learning disabilities. The identified regions encompassed the frontoparietal network commonly implicated in arithmetic processing (Rykhlevskaia et al., 2009; Peters and De Smedt, 2017; Arsalidou et al., 2018; Popescu et al., 2019); the salience network necessary for maintaining attentional focus and network switching (Ünal et al., 2025); the lingual and fusiform gyrus, which represent complex visual symbols including numbers (Cohen et al., 2002; Yeo et al., 2017; Chen et al., 2019); and the hippocampus, central for associative memory formation and recall (Giovanello et al., 2004; Cho et al., 2012; Qin et al., 2014). These findings converge on recent meta-analysis documenting atypical brain activation across similar regions in mathematical learning disabilities, including DD (Tablante et al., 2023).

This distributed signature indicates that DD reflects dysfunction in multiple brain systems, consistent with systems neuroscience models of mathematical cognition (Iuculano et al., 2018; Menon and Chang, 2022; Menon et al., 2024). Rather than implicating a single region, classification patterns encompassed “domain-specific” regions anchored on the intraparietal sulcus (IPS; Price et al., 2007; Butterworth et al., 2011) and “domain-general” networks, including frontal regions associated with executive functions, working memory, and metacognition (Menon, 2016; Bellon et al., 2020). These findings suggest that arithmetic problem-solving deficits in DD cannot be reduced to a single impaired representation but instead arise from disruption across distributed systems supporting numerical magnitude processing, visual-symbolic recognition, memory encoding, and cognitive control, consistent with multicomponent accounts of DD (Fias et al., 2013; Menon, 2016; Menon and Chang, 2022; Liu et al., 2025). This pattern is consistent with evidence that mathematical processing draws on domain-general cognitive control systems shared with other skills, suggesting that DD involves dysfunction extending beyond number-specific representations (Ünal et al., 2025).

Brain–behavior analyses established direct mechanistic links between distributed neural signatures and specific latent deficits using two complementary approaches: CCA to characterize system-level brain–behavior covariation and a transformer-based TabPFN (Hollmann et al., 2025) to identify which regions support each latent cognitive process and whether their contributions differ between groups. CCA revealed robust integration between distributed neural patterns and cognitive-metacognitive profile distinguishing the groups (*R*^2^ = 0.243), with switching sensitivity and counting efficiency showing strong positive behavioral loadings, and parietal magnitude-processing regions, the hippocampus, and ventral-visual areas showing strong positive neural loadings (Fig. 5, Table 6). Complementing these findings, TabPFN revealed that the relationship between distributed neural activity and latent processes was not simply weaker in DD but directionally reversed: across switching sensitivity and counting and decomposition drift rates, regional contributions were positive or near-zero in TA children yet negative in children with DD (Fig. 6, Table 7).

This reversal helps explain why conventional univariate analyses revealed no group differences (Kwok et al., 2023): the groups differed not in how strongly individual regions activated, but in how their distributed activity mapped onto behavior. The reversal was most pronounced in the hippocampus, the only region to differ significantly across all three parameters. The hippocampal contribution to counting and decomposition efficiency is particularly noteworthy, as hippocampal engagement is typically associated with direct memory retrieval rather than procedural strategies (Cho et al., 2011; Qin et al., 2014; Supekar et al., 2021). In children with DD, these contributions were strongly negative, suggesting that disengagement of the memory system may facilitate procedural strategy execution when retrieval and procedural processes would otherwise compete. This may point to disruptions in the typical coordination between memory and procedural systems, such that hippocampal recruitment competes with, rather than scaffolds, counting and decomposition, a mechanism that may be particularly characteristic of atypical numerical development.

A parallel reversal characterized the neural basis of metacognitive control. For switching sensitivity, contributions reversed in sign between groups across nearly the entire network, encompassing frontal, parietal, and salience regions implicated in performance monitoring and adaptive control (Bellon et al., 2020; Fleur et al., 2021; Ünal et al., 2025). In TA children, coordinated engagement of these regions supported switching sensitivity to problem difficulty, whereas in DD the same regions contributed negatively, pointing to a system-wide failure to flexibly mobilize the networks governing adaptive strategy selection. Frontal executive and parietal magnitude-processing regions routinely engaged during arithmetic problem-solving (Polspoel et al., 2017; Declercq et al., 2022) thus appear to be recruited in qualitatively different ways in DD, rather than simply being underactivated. Collectively, our results indicate that DD is characterized less by localized hypo- or hyperactivation than by an altered, often inverted, mapping between distributed neural activity and the latent processes governing strategy execution and selection.

These differential associations may help inform the targeting of future intervention studies, pointing to strategy execution efficiency and adaptive strategy selection as candidate mechanisms. However, whether interventions addressing these mechanisms induce neural change in DD remains unknown, given the scarcity of neuroimaging-based intervention research in arithmetic problem-solving; establishing such links is an important next step.

An important open question is whether domain-general frontal cortex involvement is specific to DD or reflects signatures common across neurodevelopmental disorders. Mathematical and reading processing recruit shared cognitive control networks, with children showing broader engagement than adults (Ünal et al., 2025) and similar domain-general dysfunction is reported in dyslexia (Ashkenazi et al., 2013; Martin et al., 2015), suggesting frontal cortex recruitment may reflect executive demands arising whenever children struggle to deploy task-appropriate strategies. Critically, however, differential IPS involvement points to a “domain-specific” component unlikely to generalize, as IPS dysfunction is consistently linked to numerical magnitude processing (Price et al., 2007; Butterworth et al., 2011). Stratified clinical comparison groups will be needed to disentangle domain-general from DD-specific contributions.

In conclusion, our study provides the first comprehensive computational quantification of latent cognitive and metacognitive control processes governing strategy execution and selection in children with DD. By integrating Bayesian cognitive modeling with brain-wide decoding, we uncovered latent processes invisible to conventional approaches and linked neural patterns directly to cognitive mechanisms. More broadly, this integrative framework demonstrates the power of combining computational modeling with multivariate neuroimaging to understand individual variability in DD.
